# Microbes: A Hidden Treasure of Polyunsaturated Fatty Acids

**DOI:** 10.3389/fnut.2022.827837

**Published:** 2022-03-17

**Authors:** Aabid Manzoor Shah, Wu Yang, Hassan Mohamed, Yingtong Zhang, Yuanda Song

**Affiliations:** ^1^Colin Ratledge Center of Microbial Lipids, School of Agriculture Engineering and Food Sciences, Shandong University of Technology, Zibo, China; ^2^Department of Botany and Microbiology, Faculty of Science, Al-Azhar University, Assiut, Egypt; ^3^Institute of Agricultural Facilities and Equipment, Jiangsu Academy of Agricultural Sciences, Nanjing, China

**Keywords:** polyunsaturated fatty acids, novel oleaginous microbes, metabolic engineering, genome mining, agro - industrial waste conversion

## Abstract

Microbes have gained a lot of attention for their potential in producing polyunsaturated fatty acids (PUFAs). PUFAs are gaining scientific interest due to their important health-promoting effects on higher organisms including humans. The current sources of PUFAs (animal and plant) have associated limitations that have led to increased interest in microbial PUFAs as most reliable alternative source. The focus is on increasing the product value of existing oleaginous microbes or discovering new microbes by implementing new biotechnological strategies in order to compete with other sources. The multidisciplinary approaches, including metabolic engineering, high-throughput screening, tapping new microbial sources, genome-mining as well as co-culturing and elicitation for the production of PUFAs, have been considered and discussed in this review. The usage of agro-industrial wastes as alternative low-cost substrates in fermentation for high-value single-cell oil production has also been discussed. Multidisciplinary approaches combined with new technologies may help to uncover new microbial PUFA sources that may have nutraceutical and biotechnological importance.

## Introduction

**FIGURE 1:**
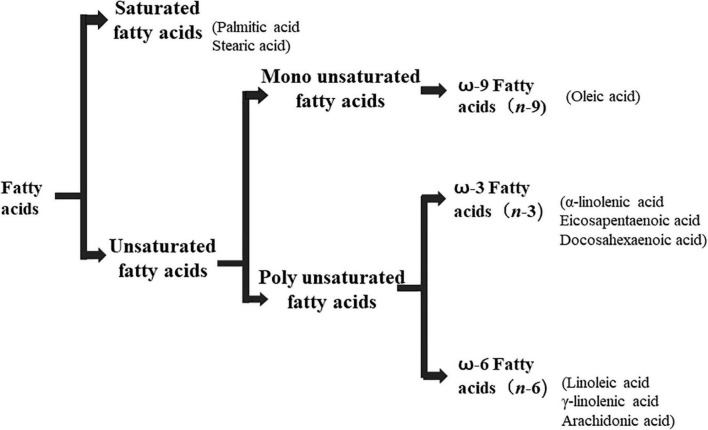
Fatty acid classification.

Lipids include oils, fats, phospholipids, steroids, waxes, and certain related compounds are characterized by water insolubility and nonpolar organic solvents solubility (1). Lipids have a biological significance for their involvement in cell membranes, energy source, signaling pathways, hormones, vitamins, gene expression, and bioactive lipid mediator synthesis (2). Lipids are an important component of the daily diet as they provide energy as well as needed fatty acids (3). Fatty acids (FAs) are the simplest lipids, organic compounds containing hydrophobic hydrocarbon chain varying in carbon atoms from C6 to C32 attached to a hydrophilic carboxyl functional group. The majority of FAs seen in nature contain linear hydrocarbon chains, while a few, mainly in bacteria, may have branching or even cyclic structures. FAs on the basis of presence or absence, number of their double bonds can be categorized into saturated fatty acids or unsaturated fatty acids (Figure 1). Dietary saturated FAs and MUFAs are mainly supplied from foods of plant and animal origin. Trans-fatty acids containing a double bond in a trans-configuration are the most common type of MUFA derived via industrial hydrogenation (4). In light of therapeutic and physiological properties, dietary polyunsaturated fatty acids (PUFAs) are regarded as being extremely significant (5). The two significant ω-6 PUFAs are arachidonic acid and linoleic acid constitute 85%, whilst the ω-3 PUFAs, such as docosahexaenoic acid, eicosapentaenoic acid, and alpha-linolenic acid constitute the remainder of the 10–15% total PUFAs (6). The human body cannot synthesize most PUFAs and they are therefore called as essential fatty acids and must be supplied through diet. On the other hand, saturated and MUFAs can be synthesized by the human body, therefore considered non-essential fatty acids (7). Fatty acid production can occur via aerobic metabolic pathways found in fungi, algae, plants, and animals, or anaerobic pathways using polyketide synthase enzymes found in some bacteria and algae. This involves both elongase and desaturase enzyme-controlled elongation and desaturation processes (8).

Polyunsaturated fatty acids play a vital role in human health, including nervous system development, cardioprotective functions, inflammatory disorders, assisting in the reduction of serum triglyceride concentrations, and cancer prevention (9). PUFAs are found in all animals and many plants, microorganisms, particularly algae, fungi, and bacteria, have the highest diversity and offers excellent alternatives for the production of nutritionally important PUFAs (5, 10). PUFAs from oleaginous microbes can be economically feasible and sustainable than animals and plants and are the target of most industrial microbial engineering (11).

**FIGURE 2:**
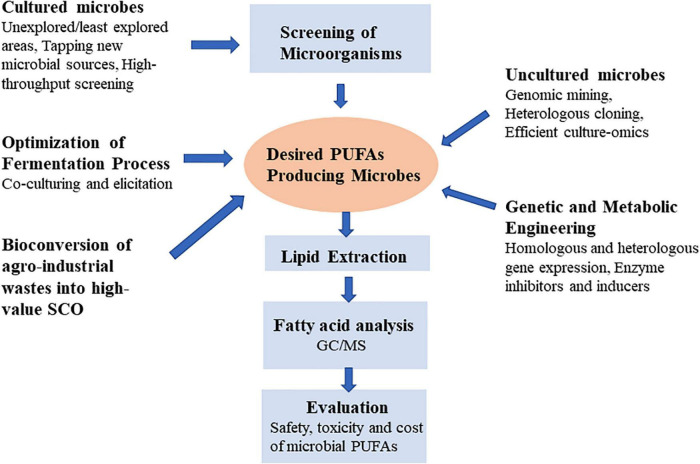
Strategies for ensuring the discovery of novel microbial PUFA production.

Lipid biotechnology is emerging as a significant subject of research due to advances in analytical techniques and genetic engineering, as well as diverse application of lipids and their derivatives in different industries. Modern lipidomics techniques, such as GC, LC, EI, MS, NMR, and MALDI can rapidly screen a large number of microbes for their lipid composition (8, 12, 13). In microbial biotechnology, the biggest problems of PUFAs production are to improve yields to meet cost competitiveness demands of different sectors (14). New perspectives for the development of sustainable microorganisms producing PUFAs would be opened up by better understanding of microbiology, analytical techniques and metabolic engineering. The benefits of study on the PUFAs in oleaginous microbes might be overwhelming from fundamental research to industrial applications. This review outlines the fundamental features of the generation of PUFA microbial cell factory. It addresses the relevance, importance, genetic modification, and novel strategies for achieving more PUFA production in microorganisms. Additionally, it also discusses how we can use more effective methodologies to find new PUFA-producing microbial strains by mining less explored natural resources (Figure 2).

## Importance of Polyunsaturated Fatty Acids

Polyunsaturated fatty acids are fatty acids with more than one double bond in their chemical structure and are categorized into n-3 (ω-3) and n-6 (ω-6) depending on where the first double bond is located on the methyl end of the FA chain. PUFAs are more commonly found in cis-form in nature. Unsaturated fatty acids in membranes are often restricted to the phospholipids’ sn-2 (sn = stereospecific numbering) position with 18 to 20 carbon atoms. PUFAs are key constituent of cell membranes, glycolipids and phospholipids, precursors for many regulatory molecules and hormones, and have been recognized as health-promoting active components as shown in Table 1 (5, 15–17).

**TABLE 1:** Details of some important PUFAs with general biological importance.

PUFA common name	Chemical structure	Systematic name	Biological importance
α-linolenic acid, (ALA), 18:3, (ω-3)	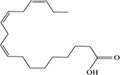	cis,cis,cis-9,12,15-octadecatrienoic acid	Important for nerve function Important for blood clotting Important for brain health Important for muscle strength
Arachidonic acid, (ARA or AA), 20:4, (ω-6)	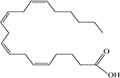	cis,cis cis cis-5,8,11,14-eicosatetraenoic acid	Preventing heart disease Pregnancy and fetal development Improve insulin sensitization Improved blood lipid profile
Dihomo-γ-linolenic acid, (DGLA), 20:3, (ω-6)	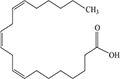	cis,cis,cis-8,11,14-eicosatrienoic acid	Avoid the progression of diabetic retinopathy Anti-angiogenic property Anti-inflammatory property
Docosahexaenoic acid, (DHA), 22:6, (ω-3)	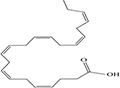	cis,cis,cis cis,cis,cis-4,7,10,13,16,19-docosahexaenoic acid	Antioxidant property Antimicrobial property Maintaining membrane fluidity Decrease obesity and associated metabolic disorders
Docosapentaenoic acid, (DPA), 22:5, (ω -3)	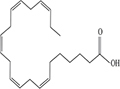	cis,cis,cis,cis,cis-7,10,13,16,19-docosapentaenoic acid	Activating brown adipose tissue aids energy expenditure Maintain normal blood glucose
Eicosapentaenoic acid, (EPA), 20:5, (ω -3)	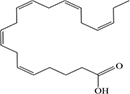	cis,cis,cis,cis,cis-5,8,11,14,17-eicosapentaenoic acid	Maintain normal Bone health Reduce LDL-Cholesterol Anticancer effects Improving hyperlipidemia
Eicosatetraenoic acid, (ETA), 20:4, (ω-3)	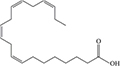	cis,cis,cis,cis-8,11,14,17-eicosatetraenoic acid	Regulation of blood pressure Regulation of cell signaling Reduce the abdominal visceral fat depot Minor antidepressant effect Antinociceptive effect
γ-linolenic acid, GLA, 18:3 (ω-6)	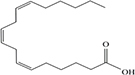	cis,cis,cis-6,9,12-octadecatrienoic acid	
Mead acid, (MA), 20:3, (ω-9)	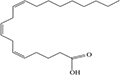	cis,cis,cis-5,8,11-eicosatrienoic acid	
Stearidonic acid, (SDA), 18:4, (ω -3)	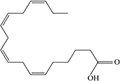	cis,cis,cis,cis-6,9,12,15,-octadecatetraenoic acid	

According to several medical studies, PUFAs are essential for brain, nerve, eye function (18, 19), prevention of neurodegenerative diseases and psychiatric disorders (20), as well as for the prevention of cardiovascular diseases (21), infection (22), and cancer (9). The American Heart Association (AHA) recommends eating oily fish twice a week for adults. Fish, particularly oily species have high levels of EPA and DHA that have been found to be cardioprotective. EPA has the main human benefits of reducing the risk of colon, breast and pancreatic cancers, lowering plasma cholesterol, protects against atherosclerosis, antiaggregatory property, protecting against cardiovascular illnesses, and is vital in maintaining homeostasis (23–26). DHA is essential for appropriate neurological and visual development in babies, as well as protection against cardiovascular disease (23, 27). GLA has also been reported to have anti-cancer properties, particularly against breast cancer and malignant glioma cells (28). Direct transformation studies on microbial lipids have resulted in the production of PUFAs salts that showed potent bioactivities than parent PUFAs. For example the lipids containing either GLA or EPA produced by *N. salina* and *T. elegans* were transformed into water-soluble fatty acid potassium salts (FAPS) and evaluated *in vitro* against a variety of human pathogenic bacteria and the MCF-7 cancer cell line. FAPS produced from the lipids were found to be potent inhibitor of various Gram positive and Gram negative bacteria, as well as breast cancer cells, even at low doses (29). Similarly, fatty acid lithium salts (FALS) derived from *C. echinulata* lipids containing GLA showed high cytotoxicity and genotoxicity inducing DNA fragmentation of HL-60 cancer cells (30). ARA performs several key physiological functions as a biogenetic precursor to the bio-active prostaglandins, prostacyclins, thromboxans, and leucotrienes (31). The Food and Agriculture Organization/World Health Organization (FAO/WHO) advised that newborn milk be supplemented with arachidonic acid (32). PUFAs also have shown antimicrobial role, inhibiting the proliferation of certain food borne and food spoilage microorganisms (33, 34). PUFAs presence in the diet improves the absorption of vitamins A, D, E, and K (fat-soluble vitamins) and also regulates cholesterol metabolism (28).

## Why Microbial Polyunsaturated Fatty Acids?

Due to the difficulty of synthetically inserting cis double bonds into saturated fatty acids, PUFAs are considered a natural product (35). According to current projections, DHA availability may decline to the point where almost 90% population would face a shortage (16). Notably, there is a significant discrepancy between demand and supply, with the deficit anticipated to be more than 1 million tons (36). Researchers throughout the world are exploring alternative sources of PUFAs and microbes have been identified as promising sources (37). Microbial production of PUFAs has attracted more attention and various companies are underway for their commercial production and marketing. In 2002, Photon Company (was founded in New Zealand to commercialize microbial EPA. Microbial PUFAs have an advantage in comparison to plant and fish oil, as microbes uses different substrates including wastes for their growth and can be cultured regardless of climate change. Microorganisms, due to their fast growth and structural simplicity, can be utilized as a model to research the PUFA metabolic pathway, leading to a better knowledge of the process and simple to manipulate in order to maximize productivity. The production via fermentation of microbial PUFAs is an excellent and renewable source. Some bacteria, fungus and marine algae are key providers of PUFAs in the microbial world (38). Numerous species of bacteria, fungi and microalgae can accumulate 20% w/w or more lipids in their cellular compartment; these organisms are known as oleaginous microorganisms (39). The majority of lipids contained in oleaginous bacteria are 4 to 28 saturated or unsaturated fatty acids of non-branched carbon chains (40, 41). Based on their fatty acid compositions, oleaginous microorganisms can be used in the biodiesel or nutraceutical industries. Oleaginous microbes can ferment low-value agro-industrial raw materials into value-added products lipids in either a solid or submerged process (42, 43). All microorganisms have the potential to synthesize lipid, but only a few of them are naturally capable of synthesizing and storing extra lipid, including PUFAs, as shown in Table 2.

**TABLE 2:** List of oleaginous microbes in nature capable of synthesizing and storing PUFAs.

PUFAs	Microbial sources	References
ARA	**Fungi** (*Mortierella alpina, Entomophthora exitalis, Blastocladiella emersonit*, and *Conidiobolus nanodes*) **Algae** (*Euglena gracilis, Sargassum salicifolium, Porphyridium cruentum, Heterocapsa tricuetr, Amphidinium* sp*., Prorocentrum minimum, Prorocentrum triestinum*, and *Tetraselmis chui*) **Bacteria** (*Phaselicystis flava*)	(44–53)
DGLA	**Fungi** (*Conidiobolus nanodes, Mortierella alpina*, and *Saprolegnia ferax*)	(44–46, 49, 54, 55)
DHA	**Fungi** (*Thraustochytrium roseum, Thraustochytrium aureum, Schyzochytrium aggregatum*, and *Schyzochytrium* SR21) **Algae** (*Crypthecodinium cohnii, microalgae* MK8805, *Gyrodinium nelsoni, Gonyaulaxpolyedra, Amphidinium carteri*, *Amphidinium* sp, *Alexandrium sanguinea, Heterocapsa tricuetra, Isochrysis galbana, Pavlova gyrans, Prorocentrum micans, Prorocentrum minimum*, and *Scripsiella trochoidea)* **Bacteria** (*Rhodopseudomonas* spp*., Vibrio* spp., and *Colwellia psychrerythraea*)	(51–53, 56–59)
DPA	**Fungi** (*Schyzochytrium* sp.)	(56, 60)
EPA	**Fungi** (*Mortierella alpina, Mortierella elongata, Pythium ultimum*, and *Pythium irregulare*) **Algae** (*Chlorella minitissima, Amphidinium* sp, *Alexandrium sanguinea, Asterionella* sp, *Chlamydomonas* sp, *Chlorella ellipsoidea, Heterosigma akasiwo, Nannochloropsis* sp, *Nitschia ovali, Pavlova gyran, Phaedacturum tricornutum*, and *Tribonema* sp) **Bacteria** (*Shewanella putrefaciens, Shewanella electrodiphila*)	(46, 47, 51–53, 56, 57, 61–64)
ETA	**Fungi** (*Mortierella alpina*)	(65, 66)
GLA	**Fungi** (*Mucor circinelloides, Mortierella isabellina, Mucor mucedo, Mortierellaramanniana, Cunninghamella elegans, Cunninghamella echinulata, Cunnin-ghamella japonica, Thamnidium elegans, Rhizopus arrhizus*, and *Gilbertella persicaria DSR1*) **Algae** (*Chlorela vulgaris, Spirulina platensis, Amphidinium* sp, *Dunaliella salina, Isochrysis galbana, Tetraselmis chui, Tetraselmis suecica*, and *Prorocentrum parvum*)	(52–54, 67–72)
MA	**Fungi** (*Mortierella alpina*)	(73)

Bacteria often produce a single type of PUFA rather than a mixture, requiring less purification and hence lowering production costs. Discovery of bacteria produces EPA and DHA from sediments of deep sea water has drawn scientific interest, which has in turn paved the way for the use of bacterial PUFAs in the nutraceutical industry (74, 75). In bacterial cells, EPA is found in the form of phospholipids such as phosphatidyl ethanolamine, phosphatidyl glycerol, and cardioolipin (76). To date, the majority of PUFA-producing bacteria have been discovered as belonging to *Photobacterium*, *Shewanella*, *Moritella*, *Colwellia*, *Psychromonas*, *Alteromonas*, and *Vibrio* (75, 77–79). It was discovered that the majority of PUFA manufacturers are topiezophilic and psychrophilic, with origins in the deep sea and polar regions (74). Increased membrane fluidity due to high PUFA content in the plasma membrane is essential for such microbes to adapt to harsh environments (74, 77).

Among fungi order Mucorales have largest number of species that are typically fast-growing, saprotrophic and many of its members were reported oleaginous (80). Zygomycete fungi produce important PUFAs such as GLA, DGLA, ARA, and EPA (81, 82). It is therefore no surprise *Mortierella alpina, Yarrowia lipolytica*, and *Mucor circinelloides* are among the best PUFA producers in the world. Even better, the list of producers is always growing. *Mucor circinelloides*, a filamentous fungus, was the first microbial strain identified to produce commercial GLA rich oil (83). *M. alpina*, an oleaginous fungus, is a good source of ARA and has been exploited commercially since late 1980s to generate ARA-rich oil for infant formula (84). *Y. lipolytica* is labeled as safe and is regarded as best host for the manufacturing of PUFAs in nutraceutical industries (84).

When it comes to producing PUFAs, microalgae are excellent source, due to their ability to thrive in the presence of nutrient- and oxygen-controlled conditions as well as carbon dioxide- and light-controlled environments (85). The photoautotrophic marine oleaginous diatom *Fistulifera solaris* has been shown to accumulate large percentage of EPA in their dry biomass (86). Diatom *Nitzschia laevis* has been reported to produce large quantities of EPA (87). *Phaeodactylum tricornutum*, a photoautotrophic diatom, produced more EPA and DHA when cultivated in a mixotrophic environment than when grown in a photoautotrophic environment. *Crypthecodinium cohnii*, a marine microalgal heterotrophic species that has been utilized for DHA production on commercial scale, is another promising candidate (88). The ability of marine microalgae to accumulate substantial amount triacylglycerides (TAGs) rich in PUFAs is remarkable. Unicellular *Thraustochytrids* such as *Schizochytrium*, *Thraustochytrium*, and *Aurantiochytrium* are important heterotrophic microalgae that accumulate significant amounts of DHA (89). Photosynthetic microalgae, like *Dunaliella salina* (90) *Nannochloropsis oceanica* (91), and *Phaeodactylum tricornutum* (92) primarily synthesize EPA (93). LA and ALA are primarily stored by other species, such as the green algae *Chlorella vulgaris* (94).

## Metabolic Engineering of Existing Microbes

In microorganisms, two separate metabolic mechanisms, desaturase/elongase or aerobic pathway and polyketide synthase (PKS) or anaerobic pathway, are responsible for the production of PUFAs (95). Several sets of genes encoded enzymes desaturase and elongase isolated and identified from the majority of eukaryotic and prokaryotic species, including animals, plants, and the majority of other microbes, showed aerobic pathway of PUFAs biosynthesis (96, 97). The D4-, D5-, D6-, D8-, D12-, and D15-desaturases have largely been found in animals, plants, and microbes (98–100). The detected elongases are classified into two groups: FAE1-like and ELO-like sequences. FAE1-like sequences are membrane-bound, and the most common plant elongases were shown to be selective for saturated and MUFAs (101). On the other hand, ELO-like sequences like ELO1, ELO2, and ELO3 are three distinct yeast elongases that have varied fatty acid substrate preferences (102). In non-photosynthetic microbes *de novo* fatty acid synthesis takes pace cytoplasm while as in the plastids of photosynthetic microbes. Enzymes for fatty acid desaturation and elongation are mostly associated with the endoplasmic reticulum membrane in eukaryotes (103).

Instead of using the desaturase/elongase pathway for PUFA biosynthesis, some prokaryotic and eukaryotic microorganisms use PKS to synthesize DHA or EPA referred as an alternative PUFA biosynthetic pathway (95). This route was first reported in *Shewanella pneumatophori* strain SCRC-2378, an EPA producing marine bacteria, which had PUFA levels ranging from 24 to 40% of total fatty acids (104). The PKS genes are thought to encode multi-functional complexes that can synthesis EPA without the assistance of FAS, aerobic desaturase/elongase. There were eight closely similar PKS proteins and three homologous bacterial FAS proteins in the protein sequences encoded by ORFs within the big DNA fragment (95). Various bacteria inhabiting in the deep sea such as *Colwellia psychrerythraea*, *Moritella marina*, *Photobacterium profundum*, and in some marine protists thraustochytrid such as *Thraustochytrium* and *Schizochytrium*, have PUFA-PKS clustered genes homologs of the Shewanella that has raised the possibility of lateral gene transfer (105–107). These homologous PKS gene clusters from PUFA-producing microorganisms varied in gene number, gene cluster order and orientation, and enzyme domain arrangement.

Metabolic engineering has been used to successfully redesign the oleaginous microbial metabolism in order to adjust the composition and properties of microbial PUFAs (108). Metabolic engineering technology interferes with parts or the entire genome, transcriptome, metabolome proteome and lipidome of microbes; therefore, understanding these pathways is essential for modifying existing strain for PUFAs rich lipid production (109). Every oleaginous microbe has unique lipid producing capabilities, and there are a variety of strategies to alter and optimize PUFA metabolic pathway (110). Researchers all over the world manipulated PUFAs synthesis, storage, and profile pathways by overexpression of genes, blocking or knockout of genes, knockdown, suppression or activation of regulators (111, 112). In natural oleaginous microbes, metabolic engineering has made it possible to increase the production of PUFAs, and in non-oleaginous microbes, it has made it possible to reconstruct PUFA biosynthesis pathways (Table 3).

**TABLE 3:** Summary of genetic engineering of different enzymes in microbes to boost the production of PUFAs.

Genes	Host organism	PUFAs	References
DHA synthesis gene cluster from *M. marina* MP-1	*E. coli* DH5a	DHA	(113)
EPA synthesis gene cluster from *Shewanella* sp. strain SCRC-2738	*E. coli* JM109	EPA	(114)
EPA synthesis gene cluster from *S. baltica* MAC1	*E. coli* EPI 300T1	EPA	(115)
*Shewanella* SCRC-2738 EPA synthesis cluster	*C. synechococcus* and *C. transconjugants*	EPA	(116)
DHA synthesis gene cluster from *Shewanella*	*L. lactis* sub sp. Cremoris	DHA	(117)
pfa gene cluster from *Aetherobacter fasciculatus* (SBSr002)	*P. putida*	DHA	(118)
D6-Des, D9-Des, D12-Des, x3-Des, D6-Elo	*S. cerevisiae*	EPA	(119)
D9-Elo, D8-Des, D5-Des, D17-Des, D12Des, CPT1	*Y. lipolytica*	EPA	(120)
D6-Des, C18/20-Elo, D5-Des, D17-Des, C20/22- Elo, D4-Des	*Y. lipolytica*	DHA	(121)
D6-1-Des, D6-2-Des, D12-Des, malce, 6pgd, g6pd	*M. circinelloides*	GLA	(122–124)
D6-Des, D12-Des, malce1, GLELO, PavD5, D17Des	*M. alpina*	ARA	(125)
D5-Elo, CoA-D6-Des, GPAT, LPAAT/AGPAT	*P. tricornutum*	EPA, DHA	(126–128)
MCAT, DGAT, D5-Des, D12-Des	*N. oceanica*	EPA	(129–131)

The discoveries of new species and early attempts to increase PUFA production have made recent advances, though they are still in the infancy stage. Studies have shown that PUFAs can be produced in heterologous bacteria such as *Escherichia coli*, *Pseudomonas putida* and lactic acid bacteria, however, the yield was very low (132). Certain barophilic and psychrophilic organisms found in the ocean depths, such as members of the *Shewanella* genus, have been demonstrated to be substantial PUFA producers (133). *Shewanella electrodiphila* MAR441 was found to produce more EPA after being supplemented with cerulenin (FAS inhibitor) (134). In *Shewanella* species PKS-like gene clusters were identified for EPA biosynthesis that was successfully expressed in *E. coli* to produce EPA (135). Heterologous expression of EPA synthesis gene cluster from *Shewanella oneidensis* MR-1 when expressed in *E. coli* XL1-Blue, low levels of EPA were reported (136). *Colwellia* strains, which are cold-adapted heterotrophs, produce DHA, which accounts for up to 17% of TFAs (137). DHA levels in *Colwellia psychrerythraea* 34H were increased with the addition of cerulenin (59). In other study DHA biosynthetic gene cluster (pfaA-D) and the pfaE gene from *M. marina* strain MP-1, improved recombinant DHA production in *E. coli* DH5a. To achieve the polyketide biosynthesis, Shewanella SCRC-2738 EPA synthesis cluster was also expressed in cyanobacteria like *Cyanobacterium synechococcus* and *Cyanobacterial transconjugants* for recombinant EPA production (116).

Several groups have reported that the biosynthetic pathways of PUFA in yeasts have been altered. *Y. lipolytica*, recognized for its potential to accumulate high lipid content rich in unsaturated fatty acids (138). Due to its non-pathogenic nature, *Y. lipolytica* has been listed in GRAS (139). Engineered oleaginous yeast, *Y. lipolytica*, was used to produce EPA for commercial purposes (120). The major composition of lipid content of wild-type strain of *Y. lipolytica* is usually PA, OA, LA, and palmitoleic acid. It was DuPont (USA) that engineered *Y. lipolytica* strain by overexpressing 20 desaturase genes, 8 elongase, 2 cholinephosphotransferase genes (total 30 copies codon-optimized genes) in combined with disruption of 2 genes involved in lipid metabolism and peroxisomal biogenesis factor that resulted in 56.6% EPA production from its TFA content (140). DuPont (USA) also engineered *Y. lipolytica* strain by overexpressing C20/22 elongase gene and Δ4 desaturase gene that resulted in the production of 18.3% DPA and 5.6% DHA (141). Overexpressing Δ6 and Δ12 desaturases from *M. alpina* resulted in the production of 20.2% GLA of TFAs in *Y. lipolytica* (121). Furthermore, the expression of bifunctional Δ12/Δ15-desaturase in *Y. lipolytica* resulted ALA production (142). A recent study found that the pfa cluster, which contains the pfa1, pfa2, pfa3, and ppt domains (myxobacterial gene) modified *Y. lipolytica* to produce high DHA (143). Recently, there has been a surge in interest in creating unusual lipids with significant industrial value. This development has been accompanied by the creation of novel synthetic biology techniques and gene editing technology for *Y. lipolytica*. The metabolic engineering of *Y. lipolytica* has produced different unusual fatty acids, such as ricinoleic acid (144, 145). The variety of lipids produced by oleaginous *Y. lipolytica* is expected to rise in the coming years to cope with the increasing demand.

*Mortierella alpina*, an oleaginous fungus, endowed with ARA found mostly in soil as a saprophyte. It should come as no surprise that strains of *M. alpina* are still being investigated extensively, with the goal of increasing ARA titres or broadening the PUFA range (146). *M. alpina* 1S-4, high accumulating ARA strain, was genetically modified to overexpress the malce1 and glelo genes, that resulted transformants to produce more ARA than the wild strain (147). A specific D17-desaturase was inserted into genome of *M. alpina* that resulted in an increased conversion of ARA into EPA (148). Heterologous expression of PPD17 another desaturase with high D17 selectivity (from *Phytophthora parasitica*) was over expressed in *M. alpina* that boosted the EPA 31.5% of total fatty acids (149).

*Mucor circinelloides*, a typical oleaginous fungus, has been extensively studied in terms of GLA production. In *M. circinelloides* the optimization was done to convert the upstream path-way intermediate oleic acid into GLA. Native desaturases capable of catalyzing the necessary conversion were overexpressed for this purpose. Homologous overexpression studies of two delta-6 (delta 6-2 and delta 6-1) and delta-12 desaturases in *M. circinelloides* increased its GLA production (122). In terms of the PUFA pathway’s downstream steps, a recent study developed the DGLA-producing mutant by heterologous expression of GLA elongase (GLELO) (150). The delta-15 desaturase gene from *M. alpina* was cloned and overexpressed in *M. circinelloides* that resulted in production of SDA (151).

*Saccharomyces cerevisiae* produces saturated FAs and MUFAs but no PUFAs (152). *S. cerevisiae* is an excellent host for eukaryotic enzymes involved in PUFA biosynthesis, such as elongases and desaturases. Recent efforts have demonstrated eicosadienoic acid, linoleic acid, and alpha-linolenic acid synthesis in recombinant *S. cerevisiae*, however, the production results is significantly less than oleaginous microorganisms (153). When high PUFAs are expressed in *S. cerevisiae* it becomes inebriated, displaying damage in their proteins, lipid peroxides, and even cell death (caspase-mediated) (154). It appears this yeast must be heavily engineered to produce significant levels of PUFAs. Low levels of EPA were also reported by co-expressing C18-PUFA specific *C. elegans* D6-elongase from and *S. cerevisiae* D6-, D5-desaturaes (155).

**FIGURE 3:**
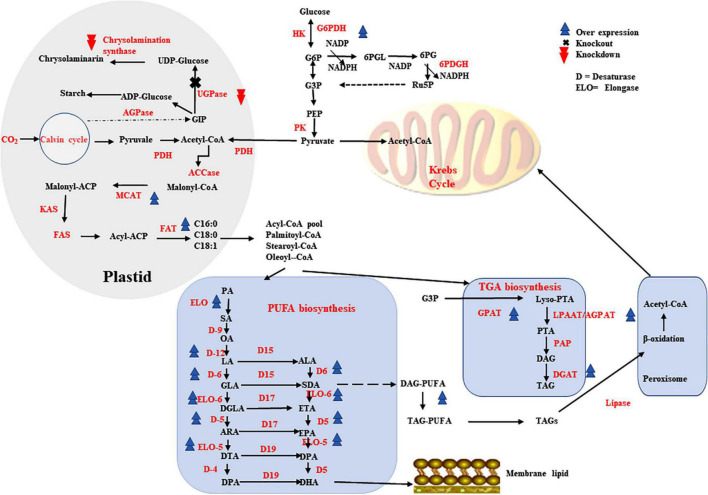
Metabolic engineering strategies that contributes higher production of PUFAs in higher microbes.

Microalgae have larger content of lipids in their dry cell mass with a variety of carbon length fatty acids, making them an excellent host for long chain PUFA production. Several microalgal species have been determined to be safe (GRAS), makes products of microalgae including PUFAs safe and widely acceptable for human food (156). In order increase PUFAs production, a number of lipid metabolism reaction fluxes, including upstream fatty acid generation and triacylglycerol biosynthesis must all be optimized as well. As various biochemical routes of lipid metabolism are interconnected, their regulation must be carefully managed (Figure 3).

Malonyl CoA-acyl carrier protein transacylase enzyme aids in the formation of malonyl-ACP during the initial phase of fatty acid biosynthesis. MCAT overexpression has been shown to be very effective in higher lipid production in a variety of microalgal species for example in *Nannochloropsis oceanica*, overexpression resulted in increased total lipid content by 31% and EPA increased by 8% (129). Another enzyme is GPAT that catalyzes glyceraldehyde 3-phosphate conversion to lysophosphatidic acid is crucial step in TAG biosynthetic pathway. When the GPAT encoding gene was overexpressed in *Phaeodactylum tricornutum*, the EPA content was increased by 40% (127). The second acyltransferase enzyme in the TAG biosynthetic route is LPAAT/AGPAT, which has narrow substrate selectivity, specifically 16–18 carbon. When the gene encoding LPAAT/AGPAT was overexpressed in *P. tricornutum* that resulted in increased content of DHA and EPA by 1.50- and 1.55-fold, respectively (128). TAG biosynthesis downstream enzymes DGAT(s) are involved in the bondage of FAs and glycerol into TAGs and also serve as excellent target for overexpression. When the DGAT gene was overexpressed in *N. oceanica*, the TAG-associated PUFAs increased by 184 %, corresponding to a 4.8-fold increase in EPA (130).

It has been discovered that overexpression of FADs and/or ELOs in microalgal cells, together with other genes showed high impact on the PUFA production. In *N. oceanica*, overexpression of individual endogenous genes encoding Δ5- or Δ12-FAD increased EPA content by about 25% (131). Overexpression of the elo1 (encodes 6-ELO) and elo2 (encodes 5-ELO) genes in *Thalassiosira pseudonana* resulted in a 12% rise in EPA and a 150% increase in DHA, respectively (157). PUFAs can be improved by overexpressing 5-FAD (PtD5b) and MCAT together, as shown in *P. tricornutum*, which accumulated large amounts of DHA, EPA and ARA (158). Overexpression of both the OtElo5 and the glucose transporter genes in *P. tricornutum* allowed the transformant to grow in glucose-supplemented medium and accumulate more DHA and EPA than the wild type under phototrophic conditions, reaching 23.6 and 36.5% of total neutral lipids, respectively (92).

*Phaeodactylum tricornutum* lipid content increased by 24% as a result of RNA interference (RNAi)-mediated UGPase gene downregulation (159). In *T. pseudonana*, chrysolaminarin synthase gene knockdown increased lipid levels up to 2.4-fold (160). The favorable benefits of downregulating a putative TAG lipase-encoding gene on increased total lipid synthesis in *P. tricornutum* were demonstrated using RNAi technology, the EPA content in TAGs increased by 70% with slightly slower growth (161). RNAi knockdown of PEPCK enzyme (phosphoenolpyruvate carboxykinase), significantly boosted lipid accumulation by roughly 40% relative to the wild type without impairing cell biomass (162). In heterotrophic marine microalgae *Aurantiochytrium sp*., G6PDH-encoding gene overexpression increased the content of LC-PUFAs, especially DPA and DHA by 25 and 12.5%, respectively (163). Endogenous Malic enzyme (ME) overexpression in *P. tricornutum* raised NADPH content by around 80% and up to twofold the production of DHA (164).

It has been proven that metabolic engineering procedures for microorganisms are helpful in enhancing PUFAs production. During nitrogen-limiting cultivation, the fundamental difference between oleaginous and non-oleaginous microbes becomes apparent. Non-oleaginous yeasts direct carbon flow into polysaccharide synthesis, but oleaginous yeasts preferentially channel it towards lipid production, resulting in an accumulation of TAGs within distinct intracellular lipid bodies. There are still numerous gaps to fill, thus more rigorous fundamental research is required to acquire a deeper understanding of oleaginous microbial biology. Specifically, it would be of interest to discover how microbes share their metabolism of lipid, including the biosynthesis of important fatty acids, and how their growth stages and environmental factors like nutrient availabilities and cultivation mode affect the lipid metabolism. Bioprocess engineering and metabolic engineering solutions should be studied simultaneously for successful industrial scale production of microbial PUFAs.

## Bioconversion of Agro-Industrial Wastes Into High-Value SCO

It has been recently brought to the attention of the scientific community that most oleaginous fungi and microalgae have the potential to grow on a variety of substrates. Agricultural raw materials used in the food industries or in industrial processing generate enormous amounts of waste each year. The availability of multiple minerals, nitrogen and carbon sources in these wastes make them appropriate as microorganism growth substrate. With the purpose of minimizing the cost of microbial lipid production, these wastes have been recommended as a substrate for oleaginous heterotrophic microbes. Furthermore, this approach should be also seen as an environmentally beneficial method of disposal of wastes (51). Oleaginous microbes that are involved in the bioconversion of agro-industrial wastes into high-value polyunsaturated fatty acids are presented in Table 4. Agricultural wastes contain a significant proportion of lignocellulosic substrates, which are mostly made of lignin, cellulose and hemicellulose, and are thus useful as carbon sources for biological transformations (165). Aquaculture wastewater is regarded as cost-effective growing media for microalgae may increase biomass yields while simultaneously removes certain nutrients from the waters, such as Nitrogen and Phosphorous (166, 167). The majority of oleaginous microbes are unable to utilize lignocellulose. The genetic manipulation of oil producing fungal strains may also allow for the direct lignocellulose conversion into SCO, a method that is previously being used in the generation of biofuels such as ethanol by using engineered yeast and fungi that are capable of assimilation of lignocellulosic materials (168). There is also another possibility to do genetic manipulation of lignocellulolytic microbes that enable them direct convert lignocellulosic substrates to lipids rich in PUFAs. *Trichosporon oleaginosus*, an oleaginous basidiomycete yeast well-known for its capacity to exploit xylose-containing substrates, has been modified to manufacture exceptionally LCFA, such as eicosatrienoic acid and eicosadienoic acid, which are intermediates in the production of the EPA and DHA (169).

**TABLE 4:** Summary of bioconversion of agro-industrial wastes into high-value single cell oil by oleaginous microbes.

Microorganisms	Substrate	PUFAs	References
*M. isabellina*	Corn stover and douglas fir forest waste	GLA	(174, 175)
*M. isabellina*	Sweet sorghum and rice hull hydrolysates	GLA	(176)
*C. echinulata*	Waste from edible oil plants	GLA	(177)
*C. echinulata*	Potato industry starch waste	GLA	(178)
*C. echinulata*	Tomato hydrolysate enriched with glucose	GLA	(179)
*M. recurvus*	Hydrolyzed molasses	GLA	(180)
*M. circinelloides* and *M. isabellina*	Liquid waste from the cheese-making process	GLA	(181, 182)
*T. elegans* and *C. echinulata*	Sugar-based and corn steep lignocellulosic wastes	GLA	(176, 183)
*Schizochytrium* sp. DT3	*Cannabis sativa* (hemp) biomass	DHA	(184)
*P. tricornutum*	Birch and spruce biomass	EPA, DHA	(185)
*Trichosporon oleaginosus*	Xylose-containing substrates	ETA, EDA	(169)
*Aurantiochytrium* sp., and *Schizochytrium* sp.	Sugarcane bagasse hydrolysate	DHA	(186, 187)
*Schizochytrium mangrovei*	Coconut water	DHA	(188)
*Aurantiochytrium* sp	Orange peel extracts	DHA	(189)
*Aurantiochytrium* sp	By-product of the palm oil industry	DHA	(190)
*T. kinney and S. limacinum*	Crude glycerol		(191, 192)
*T. elegans, U. isabellina, C. echinulata, U. ramanniana, Z. moelleri* and *Mucor* sp	Crude glycerol	GLA	(193–197)
*Mortierella* spp	Crude glycerol	ARA, DGLA	(198)
*N. gaditana*	Phosphate-limited wastewaters obtained an aquaculture station	EPA	(199)
*Navicula* sp*., Chlorella* sp*., Nannochloropsis* sp*., Dunaliella* sp., and *Tetraselmis* sp.	Shrimp farm wastewaters	EPA	(200)
*Euglena gracilis* and *Selenastrum* sp.	Fish farm wastewater	ALA, ARA, EPA, DHA	(166)
*Mortierella* sp	Shrimp shell wastes	ARA, DGLA	(201, 202)
*Mucor circinelloides* and *Rhizopus* sp	Cooking oil (Soybean, Sesame, Canola, Sunflower, Palm)	GLA	(203, 204)
*C. echinulata*	Corn steep liquor, and soybean oil waste	GLA	(205)
*Mortierella alpina* CFR-GV15	Linseed oil and garden cress oil	ALA, EPA and DHA	(206)

Finding suitable substrates is one of the most important factors in large-scale SCO production, and oleaginous microorganisms can ferment both hydrophilic and hydrophobic substrates. However, lipid fermentation on hydrophilic and hydrophobic substrates follows two distinct bio-pathways: “*de novo*” and “*ex novo*.” It is worth noting that both pathway of lipid fermentation have advantages: “*de novo*” lipid fermentation typically produces a large amount of lipid, whereas “*ex novo*” lipid fermentation can transform the extracellular and intracellular lipid composition to meet the needs of the food or chemical industries. The ability of microorganisms to use glucose, xylose, arabinose, etc. as carbon sources is highly desired in order to increase the efficiency of biomass and lipid production, while an important drawback associated with the lipid fermentative conversion of sugar-based residues is the production of other metabolites such as intracellular polysaccharides, extracellular citric acid. For example under nitrogen-limited conditions, *M. isabellina* ATHUM 2935 cultivated on glucose/xylose blends, demonstrated remarkably different physiological and biochemical patterns related to the sugar used as substrate. When glucose-rich media were used as carbon sources, significant lipid production occurred, while endopolysaccharides and polyols (mannitol and xylitol) were also produced in varying amounts depending on the substrate composition (170).

There have been reports of oleaginous microorganisms assimilation of hydrophobic substrates such as esters, TAGs, FAs, sterols, etc. via distinct pathway namely ex novo lipid synthesis (170). Hydrophobic materials acquired from an industrial waste stream, waste cooking oils, effluents from dairy and butter producing enterprises, and waste fish oils can all be used as hydrophobic feedstock for biomass and lipid production by oleaginous microbes (171). *Y. lipolytica* has been widely investigated in an effort to understand how hydrophobic substrates are utilized by the organism (172). The active transport of hydrophobic substrates into the cell is facilitated by secreted lipases, therefore only the microorganisms capable of producing lipases can incorporate free FAs. Once inside the cell, free FAs can be used for energy or they can be integrated into lipidic structures for storage. Fat biomodification by oleaginous microbes can be used to tailor the FAs profiles of hydrophobic substrates into value-added oils (173). It is common for hydrophobic substrates to form layers on the surface of the water, which prevents the oleaginous yeast from accessing them during fermentation experiments. Despite this, some oleaginous yeast can produce extracellular emulsifiers that can reduce the size of hydrophobic substrates and improve their miscibility in water. To make hydrophobic substrates more accessible to oleaginous microorganisms, researchers sometimes added emulsifiers like Tween 80, Span 80, Tween 60, and OP-10 to the aqueous medium. The addition of emulsifiers to the fermenting medium, on the other hand, raises the overall production cost and has an impact on the final lipid production because it is frequently dissolved in organic solvents, interfering with the estimation of residual hydrophobic substrates.

## Approaches That Leads Novel Sources of Microbial Polyunsaturated Fatty Acids

### Tapping New Microbial Sources

Based on DNA analysis and mathematical modeling, it is widely acknowledged that just a small fraction of microbial diversity has been studied, and that even the majority of microbial species have yet to be isolated and tested for chemical diversity (72, 207). Unusual habitats surveys have produced numerous novel microbes with important fatty acids in the past, hence unknown microbial species occupying rare habitats with various environmental restrictions are regarded resources for PUFA production (72, 208–211). Underexplored ecosystems include hyperarid, high-saline environments desert, forest as well as unique microbes that coexist with higher species in symbiotic/mutualistic interactions. With this strategy, a new PUFA microbial source has already been discovered (75, 212). The oceans, which cover more than 70% of the Earth’s surface, are a rich source of microorganisms with different morphological, physiological, and chemical diversity. Considering that just 7–8% of oceans are made up of coastal areas, the deep-sea biosphere, which extends from the seashore to depths of >10,000 m, contains 95% of the ocean’s microorganism biomass and is a potential source for novel microbial metabolites (213). The sampling of deep ocean sediments remains one of the obstacles in harnessing new source of microorganisms. Access to the deep ocean is generally dependent on conventional oceanographic processes that are not specifically developed for microbiological research, which excludes huge areas of the ocean floor from sampling range (214). The Antarctic marine environment is home to cold-adapted prokaryotic and eukaryotic species with unique characters that allow them to survive in saltwater at a constant low temperature. A number of Antarctic microorganisms have shown an increase in fatty acid unsaturation with decreasing growing temperature (76). It is necessary for deep-sea bacteria to thrive in a psychrophilic and piezophilic habitat for EPA to take effect (210). *Shewanella livingstonensis* Ac10, for example, is a psychrotrophic bacteria isolated from Antarctic saltwater that produces a considerable amount of EPA (Kawamoto et al. 2009). There is a marine bacterium known as *Shewanella piezotolerans* WP3 that has been identified as psychrotolerant and piezotolerant (75). It was found in sediment from the western Pacific Ocean at a depth of 1914 m, where it thrived at temperatures and pressures as high as 20 MPa. EPA is found in the membrane of *S. piezotolerans* cells (215, 216). At a depth of 5110 meters, the Ryukyu Trench was home to the discovery of *Shewanella violacea* strain DSS12. At 8 °C, and 30 MPa, it thrives under a moderately piezophilic environment. Even at 0.1 MPa, it can grow at 8°C, *S. violacea* DSS12 cells have a 15% EPA membrane content (216, 217). Several marine bacterial cultures capable of producing ω-3 FAs have been isolated in recent years, and the metabolic pathways involved in their synthesis have been identified. It has been recognized that the majority of known microbial producers are gamma-Proteobacteria, specifically those belonging to the genera *Shewanella*, *Photobacteria*, *Colwellia, Vibrio, Psychromonas*, and *Moritella* (76, 218). Some isolates associated with Psychroserpens and Flexibacter in the Flavobacteria class have also showed the ability to synthesize ω-3 LC-PUFAs (75). Several thraustochytrid strains have been isolated from seawater (219–221). Thraustochytrids are well-known for producing important ω-3 PUFAs like EPA, and DHA (222). Thraustochytrids may store lipids in excess of 50% of their dry weight, with DHA accounting for more than 25% of TFAs (212).

### Cultivating Rare Microbes

The importance of rare microorganisms in microbial communities is still a topic of debate. Scientists estimate that there are at least 10 million different kinds of microorganisms in the world, and DNA data shows that just 1% of those microorganisms can be easily cultivated in a lab (223, 224). This is due to the fact that many rare microbes are slow-growing, some may have specialized requirements. Many rare microbes that require special media have been isolated by changing media conditions, such as utilizing seawater-based isolation medium to allow marine organisms to grow (225). Signaling molecules like homoserine lactones, cAMP and pyruvate been demonstrated to increase the number of microorganisms from various habitats and assist uncultured strains recover (226). This type of approach was found to be very successful in isolating microbes that produced various bioactive secondary metabolites. However, as far as microbes that produce PUFA are concerned, there has been no such report till date. But this approach can’t be underestimated in exploring the microbial world for the production of PUFAs.

### High-Throughput Screening

High-throughput screening approaches use automated screening and large-scale data analysis to speed up the discovery of new oleaginous microorganism sources. HTS is very useful in the screening process of strain libraries for microbial metabolites (227, 228). Because of the miniaturization of fermentation technologies, it is now possible to test a large number of strains under carefully regulated conditions. The flexibility to work with a variety of strains and targeted models is one of the benefits of HTS, as it saves both time and resources. Till date, a number of oleaginous yeasts have been investigated, including Basidiomycota and Ascomyta, despite the fact that the majority of genera and species have yet to be tested for oil content (229–231). One significant factor to remember is that yeast taxonomy is always changing, therefore when determining whether a yeast species is oleaginous or not, special attention should be paid to identification of species. To quantify lipids generated by microorganisms, standard technologies such as HPLC, GC, TLC, and MS are available. Although these methods are highly reliable and credible, they require expensive and heavy equipment, making large-scale yeast screens time-consuming and difficult (232, 233). A standard lipid extraction method must be developed for all oleaginous microorganisms in order to avoid the use of harmful organic solvents and other drawbacks associated with previous methods for quantification in various biological models. Numerous approaches for quick screening have been proposed in the past like fluorimetric methods using Sudan black (234), Nile red (235), and Nile blue (236) and, presently the use of FTIR (237). Following this basic screening, more robust and thorough methods for confirming and validating the results should be applied. HTS tools for assessing fatty acid composition in oleaginous yeast and microalgae species are currently being used, with promising results (238).

### Co-culturing and Elicitation

Under normal laboratory fermentation conditions, many putative gene clusters can go unnoticed or unexpressed making the production of microbial PUFAs via established conventional routes difficult. The major research on microbial oil has been done on single cell cultures. New methods for activating silent genes that involve the co-culture (two or more microorganisms are cultivated together in the same environment) are gaining popularity. In the co-culturing method, producer strain released the products by the activation of molecules or precursors secreted by inducer strain. Because of the interactions between diverse cultures that present in most natural environment, this system has been regarded as an effective model. Mostly microbes are frequently found in intimate connection with one another; they live and grow in the same biocoenosis for as long as source of nutrient are accessible. As a result, microbial interactions between cultures might be exploited and reproduced at the laboratory scale. This is not a new concept; for decades, mixed cultures have played a key role in wastewater treatment, creating biomass and bioactive chemicals production (239–241). Elicitation involves presenting microbial cells with a variety of signals, and the use of these so-called ‘elicitors’ has been shown to be effective in the production of various new metabolites (242, 243). When microorganisms detect these external signals, they produce discrete reactions that result in a changed metabolite profile, and if induced chemicals are precisely targeted, new structures are expected.

To attain much better lipid productivity, scientists are using a co-cultivation technique that attempts to reconcile the discrepancy between biomass productivity and lipid content. Various studies have been conducted to improve lipid productivity by encouraging the accumulation of total biomass and lipid output through the use of a co-culture strategy with various microbial species. In general, research on this method has been described for algae species e.g., the co-culture of *C. sorokiniana* and *Chlorella vulgaris* with *Azospirillum brasilense* increased not only lipid, but also variety of fatty acids, (244). Many other species have been reported to have high growth coupled with other microbial species, such *Rhodotorula glutinis* and *Ambrosiozyma cicatricose* (245–247) and *Monoraphidium sp* FXY10 (248, 249), Dostalek found that the co-culture of *R. toruloides and Saccharomycopsis fibuligera* in starch media resulted in a greater lipid and biomass content (250).

### Genome-Mining Approaches

The rapid move from single-gene to entire-genome analysis was made possible by advances in sequencing technology, which provide yet another significant platform for revealing microorganisms’ potential (242). Diverse putative gene clusters remain silent under various conditions, making it challenging to discover their entire metabolic potential. HTS can aid in the discovery of silent gene clusters in microbial genomes, as well as the subsequent characterization of cryptic products using heterologous gene expression or gene knockdown experiments combined (251, 252). When a species’ genetic information is insufficient, such as in many “non-model” organisms, *de novo* genome assembly makes it possible to do functional genomics research on them. It was discovered that the PKS pathway, which is responsible for the manufacture of DHA, requires sufficient NADPH and acetyl-CoA, and that 30 genes were anticipated to be possible targets for DHA overproduction in *Schizochytrium limacinum* SR21 (253). Two newly identified Thraustochytrids strains, SW8 and Mn4, had their genomes dramatically devoured by secreted carbohydrate-active enzymes, as revealed by comparative genomic research. The FAS and PKS routes of PUFA generation were found to be incomplete (254). *Thraustochytrium sp.* 26185’s genome sequence and analysis also revealed the presence of anaerobic and aerobic PUFAs metabolic pathways. But, the aerobic route lacked a crucial gene for stearate delta-9 desaturase. Although the genome of NP10 was found to be extremely similar to that of numerous strains in the *S. griseus* clade, comparative research targeted at identifying potential FA synthases assisted in the discovery of a prospective gene cluster. Following gene inactivation and complementation, it was discovered that this cluster was involved in the manufacture of a variety of FFAs in the NP10 organism (255). This genome mining strategy resulted in the putative pfa gene cluster identification in a number of *S. cellulosum* strains. A fatty acid methylesters analysis by GC-MS revealed that in these strains’ PUFA synthesis is limited to eicosadienoic acid (EDA) and linoleic acid (LA) (256). Additionally, from the genus Aetherobacter, myxobacterial isolates have been found to be prolific makers of LC- PUFAs. Using FAME, these strains were found to produce AA, EPA, and DHA (257, 258). Anaerobic polyunsaturated fatty acid (PUFA) production via PKS-like and FAS-like synthases was discovered in many terrestrial myxobacteria of the Sorangiineae suborder (259). The LC- PUFAs EPA and ARA were also found in the *Enhygromyxa salina* SHK-1T member of Nannocystineae (260, 261). Genome mining of the closely related strains *E. salina* SWB005, *E. salina* SWB007 *E.* and *salina* DSM 15201, using publically available genome sequences also indicated pfa gene clusters presence that was identical to known myxobacterial pfa clusters (262).

## Genomic Analysis

A thorough picture of lipid metabolism in oleaginous microorganisms was provided by genome analysis, route mapping, and major lipid profiling. It will be critical to understand how gene expression is regulated during lipogenesis in various oleaginous microorganisms, as well as how it is coordinated between the creation of precursor molecules like pyruvate and acetyl-CoA, the production of the reductant NADPH, and the synthesis of lipids.

The *M. alpina* genome is 38.38 Mb in size and contains a significant number of gene duplications. More than half of its 12,796 gene models and 60% of the estimated lipogenesis pathway’s genes are part of multigene families. *M. alpina’s* fatty acid synthase is a single polypeptide that contains all of the catalytic domains required for fatty acid whereas this enzyme is composed of two polypeptides in many fungi. According to phylogenetic study based on genes involved in lipid metabolism, oleaginous fungus may have gained their lipogenic capacity throughout evolution following the divergence of Ascomycota, Basidiomycota, Chytridiomycota, and Mucoromycota (229). FADS12 and FADS15 from *M. alpina* catalyze the production of omega-6 and omega-3 polyunsaturated fatty acids, respectively. More than half of the total fat in *M. alpina* was composed of AA, which is synthesized at a much higher rate than EPA. Because there is just one gene for each desaturase in the *M. alpina* genome, this difference in capacity cannot be explained by gene duplication. (229). However, it is probable that the difference is due to the expression levels or catalytic activity of FADS12 and FADS15, as forced expression of FADS15 in *M. alpina* 1S-4 significantly boosts EPA synthesis (263).

Comparative genomics approach facilitates the identification of multiple differently expressed genes between distinct strains of the same microorganism (264). The genome of *M. circinelloides* CBS 277.49, a low lipid generating strain, was compared to *M. circinelloides* WJ11, a high lipid generating strain. The similarities between CBS 277.49 and WJ11 suggested that they are similar in terms of gene identity and gene order. The WJ11 G+C content and size of genome assembly were found to be about 39% and 35.4 Mb, respectively. it has also been revealed that the genomes of the two strains have numerous homologous regions and these regions were mostly co-linear. The number of genes for lipid accumulation enzymes was compared via reorganization of lipid metabolism and central carbon routes (265). There are two genes encoding Δ9 desaturase in both strains. As *M. circinelloides* is a GLA-producing filamentous fungus, as expected, in both strains, there were two genes encoding fatty acid delta-12 desaturase (Δ12) to convert oleic acid to linoleic acid and one gene encoding fatty acid delta-6 desaturase (Δ6) to convert linoleic acid into γ-linolenic acid (265). The majority of enzymes in the PPP pathway were encoded by the same number of genes in WJ11 and CBS 277.49, however, in WJ11, G6PDH was encoded by four genes, but in CBS it was encoded by three genes. More genes encoding G6PDH may indicate that WJ11 has more opportunity to create NADPH for fatty acid biosynthesis (265). Some enzymes in the TCA cycle, such as succinyl-CoA ligase (SUCLG), succinate dehydrogenase (SDH), and fumarate hydratase (FH), were encoded by fewer genes in WJ11 as compared to CBS strain, indicating a more active TCA cycle in CBS that in turn has the inverse relationship to fatty acid accumulation (265).

## Safety, Toxicity and Cost of Microbial Polyunsaturated Fatty Acids

Triacylglycerides and sterols, which are identical to those found in plant and animal oils, are the major components of microbial oils, with the exception of bacterial oils. Microbial oils, however, are considered new foods due to their origin, and certain regulatory hurdles must be passed before they can be utilized as food. Microbial oils have been examined for their safety, nutritional value, and clinical usefulness for human use. It is critical to study the producing organism while analyzing the safety of any microbial oil. There should not have a history of creating hazardous metabolites, nor should it be capable of infecting animals or harming the environment. In the absence of any doubt, pathogenic organisms should not be taken into account, as there is always the possibility of live organisms escaping from a production facility to infect surroundings. Oils may thus be shown to have extremely minimal non-lipid content and so cannot contain any substantial amount of any element that is likely to be dangerous (266). When oil was introduced from a new source, such as the first SCO from *M. circinelloides*, *M. alpina, C. cohnii*, and *Schizochytrium sp* which all represented “new” sources of oil at the time of their introduction, regulatory authorities required complete dossiers of evidence for the safety of these oils. In fact, the oil was purer than other plant oils, as herbicide and pesticide residues were virtually missing from the microbial oil. An important factor in this decision was that the producing organism had already received GRAS (Generally Recognized As Safe) certification. Microbial oils have been shown to be safe, but this evidence must be evaluated by various regulatory bodies around the world: The FDA in the United States; the EU ‘Regulation on novel foods and novel food ingredients’; the Canadian Food and Drugs Regulation; the Australian/New Zealand Food Standards Code; etc. These regulatory bodies are ultimately responsible for approving the sale of new foods and nutraceuticals. In the case of *M. circinelloides*, preliminary toxicity tests were carried out with brine shrimps, followed by 90-day animal feeding studies with several rats and other animals. All studies were fully successful, and the data was subsequently given to the UK Advisory Committee on Novel Foods and Processes for review and approval. There were no problems to the oil being sold in the UK (266). Since it has now been two decades since microbial oils have been consumed by infants, adults, and a wide range of animals, including fish and poultry. There has been no substantiated report that there has been a problem with their consumption, and the SCOs’ manufacturers and distributors have no reason to doubt their products’ complete safety and reliability. The safety of DHASCO™ (high DHA microbial oil) and ARASCO™ (high ARA microbial oil) in newborn formulae has been shown in numerous non-clinical and clinical investigations.

The cost of microbial oil is mostly determined by the species used for cultivation, cell biomass and the concentration of lipids within cells (267–271). Table 5 compares the amount of accumulated oil in some selected oleaginous microbes and plants. Oil production of microbes is generally expressed in kg/m^3^/year, whereas plant oil productivity is expressed in kg/ha/year. Jones et al. found that growing *C. curvatus* in a 30–50 m^3^ fermenter gives the same amount of oil as 150 palm trees in a 1-ha area in a year (271).

**TABLE 5:** Lipid productivity comparison of some selected oleaginous microbes and plants. Microbial oil is generally expressed in kg/m3/year, whereas plant oil productivity is expressed in kg/ha/year.

Source (Microbe and plant)	Oil yield (kg/m^3^/year)	Oil yield (kg/ha/year)
*R. toruloides*	2120	–
*C. curvatus*	1154	–
*M. isabellina*	679	
*C. echinulata*	134	
*C. gracilis*	525	–
*S. limacinum*	404	–
Sunflower	–	500–700
Soybean	–	450–500
Palm	–	3000–5000

Only a few techno-economic studies have addressed the prices of microbial oil that ranges between $1.72 and $5.9/kg as compared to plant oils which are sold between $ 0.5 and 1.9/kg (270). 60 to 75 % of the total price of the microbial oil is related to the cost of the substrates needed by microbes to produce the lipids. Using an effective strain and low-cost substrate can therefore help to increase the profitability of single-cell oil production.

## Future Perspectives and Concluding Remarks

There is a limited supply of polyunsaturated fatty acids on the commercial market, so scientists around the world are attempting to discover and develop new strains of organisms with essential PUFA profiles. Microbes have gained a lot of attention for their potential when it comes to producing PUFAs, but only a small percentage of them have been cultured so far. The combination of genomic platforms with high-throughput screening and fermentation studies could reveal hidden microbial’ PUFAs producing potential. It’s worth noting that oil production via fermentative procedures using microorganisms is quite costly. Greater attention must be paid to the extraction of oils from novel or genetically engineered species. The high cost of microbial oil production must be solved by utilizing low-cost carbon and other nutrients for growth, as well as a low-cost harvesting approach for biomass recovery and a superior lipids extraction procedure. Bio refinery techniques for PUFAs production, for total exploitation of microbial oils, will, in addition to other pathways, address the challenges related with PUFAs production cost. We should keep looking for novel PUFA-rich microorganisms from nature that can make a contribution to this sector. Multidisciplinary initiatives integrating microbiologists, molecular biologists, and natural product chemists are necessary to expedite PUFA generation from microorganisms.

## Author Contributions

AS and YZ performed literature research and wrote manuscript draft. WY and HM performed fact checking and finalized table and figures. YS designed and revised the final draft. All authors contributed to the article and approved the submitted version.

## Conflict of Interest

The authors declare that the research was conducted in the absence of any commercial or financial relationships that could be construed as a potential conflict of interest.

## Publisher’s Note

All claims expressed in this article are solely those of the authors and do not necessarily represent those of their affiliated organizations, or those of the publisher, the editors and the reviewers. Any product that may be evaluated in this article, or claim that may be made by its manufacturer, is not guaranteed or endorsed by the publisher.

## Abbreviations

PUFAs: polyunsaturated fatty acids
SCO: single-cell oil
HTS: high-throughput screening
FAs: fatty acids
C: carbon
MUFAs: monounsaturated fatty acids
ω: omega
LC: liquid chromatography
GC: gas chromatography
EI: electrospray ionization
MS: mass spectrometry
NMR: nuclear magnetic resonance
MALDI: matrix-assisted laser desorption/ionization
ALA: α -linolenic acid
ARA: arachidonic acid
DGLA: dihomo- γ -linolenic acid
DHA: docosahexaenoic acid
DPA: docosapentaenoic acid
EPA: eicosapentaenoic acid
GLA: γ -linolenic acid
MA: mead acid
SDA: stearidonic acid
TAGs: triacylglycerides
PKS: polyketide synthase
GRAS: generally regarded as safe
PA: palmitic acid
OA: oleic acid
LA: linoleic acid
TFAs: total fatty acids
MCAT: malonyl CoA-acyl carrier protein transacylase
GPAT: Glyceraldehyde 3-phosphate acyltransferase
LPAAT: Lysophosphatidate acyltransferase
DGAT(s): Diacylglycerol acyltransferases
PEPCK: phosphoenolpyruvate carboxykinase
G6PDH: glucose-6-phosphate dehydrogenase
LCFA: long chain fatty acids
HPLC: high-performance liquid chromatography
TLC: thin-layer chromatography
FTIR: fourier transform Infrared spectroscopy.
